# Starvation-Induced Dietary Behaviour in *Drosophila melanogaster* Larvae and Adults

**DOI:** 10.1038/srep14285

**Published:** 2015-09-24

**Authors:** Muhammad Ahmad, Safee Ullah Chaudhary, Ahmed Jawaad Afzal, Muhammad Tariq

**Affiliations:** 1 Department of Biology, SBA School of Science and Engineering, Lahore University of Management Sciences, Sector-U, DHA, Lahore, 54792, Pakistan

## Abstract

*Drosophila melanogaster* larvae are classified as herbivores and known to feed on non-carnivorous diet under normal conditions. However, when nutritionally challenged these larvae exhibit cannibalistic behaviour by consuming a diet composed of larger conspecifics. Herein, we report that cannibalism in *Drosophila* larvae is confined not only to scavenging on conspecifics that are larger in size, but also on their eggs. Moreover, such cannibalistic larvae develop as normally as those grown on standard cornmeal medium. When stressed, *Drosophila melanogaster* larvae can also consume a carnivorous diet derived from carcasses of organisms belonging to diverse taxonomic groups, including *Musca domestica*, *Apis mellifera*, and *Lycosidae sp*. While adults are ill-equipped to devour conspecific carcasses, they selectively oviposit on them and also consume damaged cadavers of conspecifics. Thus, our results suggest that nutritionally stressed *Drosophila* show distinct as well as unusual feeding behaviours that can be classified as detritivorous, cannibalistic and/or carnivorous.

Unlike mammals, where the foetus develops in the mother’s womb, fertilized eggs of insects develop outside the female body^1^. All insects go through a larval phase of development that precedes metamorphosis^2^. Soon after hatching, larvae forage for nutrients in their vicinity and eventually find a favourable niche that contains an adequate supply of food. This phase of insect development is dedicated to feeding voraciously, and fulfils the much-needed food requirements for the subsequent non-feeding pupal phase. Ingested nutrients are stored as fat bodies^3^,^4^ which serve as potential energy reservoirs for synthesis of macromolecules^5^ that are essential for cellular growth during larval and pupal stages^5^.

*Drosophila melanogaster* has emerged as a tractable model organism for studying a number of biological phenomena including feeding behaviours under various conditions^6^. Considered as a herbivore, *Drosophila* primarily thrive on vegetative matter decomposed by microbes^7^. At times, *Drosophila* larvae may encounter nutritional stress that could be transient or chronic and therefore must learn to adapt to it for survival. Remarkably, this species has evolved to adapt to ephemeral as well as persistent nutritional stress^8^,^9^. An earlier report suggested that larval malnutrition significantly affects foraging behaviour in *Drosophila*^10^. One of the striking behaviours exhibited by nutritionally challenged *Drosophila melanogaster* larvae is cannibalism, where they begin to feed on their own kind^11^. Younger larvae predate and consume wandering third instar conspecific larvae showing that cannibalism in *Drosophila melanogaster* larvae entails attack and consumption of conspecifics that are larger in size. Existence of cannibalism in *Drosophila melanogaster* adults has also been investigated^12^. Importantly, there was no difference in the death time (i.e., time until death of starving adults) between flies given access to carcasses of conspecific flies compared to ones kept under starved conditions. The authors hence concluded that cannibalistic diet does not extend fly lifespan.

Herein we report that *Drosophila melanogaster* larvae, when placed under nutritional stress, not only scavenge on larger conspecifics as reported previously^11^ but also become efficient egg predators. Additionally, our experiments suggest that carnivorous behaviour in *Drosophila melanogaster* is not only limited to feeding on its own kind, but also includes consumption of cadavers from a variety of organisms belonging to diverse taxa. Our study also shows that adult flies fail to feed on intact conspecific carcasses but they selectively oviposit on these conspecific carcasses. Moreover, *Drosophila* adults can effectively consume cut or damaged conspecific carcasses since they are ill-equipped to puncture into them. Taken together, our data suggest that adult files are capable of sensing and evaluating the nutritional content in conspecific carcasses, and under starvation conditions are able to fulfil their nutritional needs from a wide array of sources.

## Results

### *Drosophila melanogaster* larvae can effectively predate on conspecific eggs

**Figure 1:**
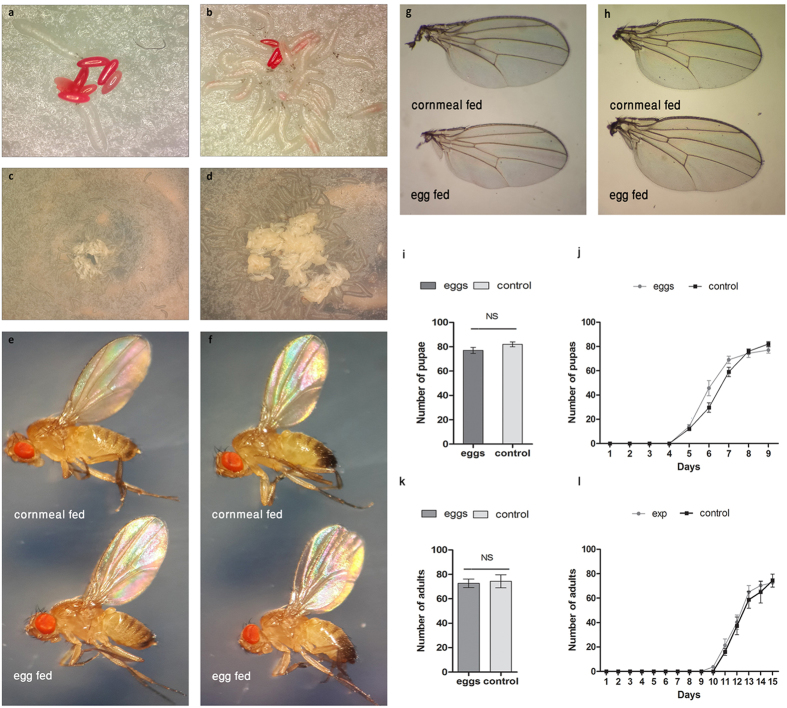
Egg cannibalism in *Drosophila melanogaster* larvae. (**a**) *Drosophila* eggs stained with red dye offered to starved Canton-S larvae. (**b**) Larval aggregation, egg consumption and dye accumulation in larval gut after 30 minutes. (**c**,**d**) Larvae tested for cannibalistic behavior with presentation of unstained conspecific eggs. *Drosophila* female (**e**) and male (**f**) adults developed by consuming conspecifics eggs and standard cornmeal-medium showed no morphological differences. Anatomical structures (i.e. head, eyes, wings, legs, thorax and abdomen), of flies developed normally after consuming eggs. Female (**g**) and male (**h**) wings of *Drosophila* reared exclusively on eggs or cornmeal, show no difference in the venation patterns (**i**) Larval development into pupae after feeding exclusively on conspecific eggs. Numbers are out of 100 larvae (mean ± s.e.m.) which successfully pupated by consuming eggs versus cornmeal. No significant difference was observed in the number of pupae emerging from larvae in either case (P = 0.4839), using Fisher’s exact test. (**j**) Cumulative number of pupae (mean ± s.e.m.) emerging from larvae fed on eggs versus cornmeal. No significant difference was observed in developmental time (P = 0.9805), using Mann-Whitney test. (**k**) Pupal development into adults after feeding exclusively on conspecifics eggs versus cornmeal. Number of adults (out of 100) which successfully eclosed (mean ± s.e.m), versus those which eclosed after feeding on standard cornmeal medium. No significant difference was observed in the number of adults in either case (P = 0.8736), using Fisher’s exact test. (**l**) Cumulative number of adults (mean ± s.e.m.) eclosed after larval feeding on eggs versus cornmeal medium. No significant difference was observed in developmental time (P = 0.733), using Mann-Whitney test.

For analysing cannibalism in *Drosophila* larvae under nutritional stress, we developed an assay in which eggs were provided as the only food source to foraging larvae reared on nutrition deficient media (see Methods). To determine whether the content ingested by cannibal larvae was derived from the conspecific eggs, fly eggs were stained with food dye and presented to starved larvae (Fig. 1a and Supplementary Fig. 1a–d). This exercise revealed that starved *Drosophila* larvae aggregated around conspecific eggs within thirty minutes and consumed them as demonstrated by the accumulation of dye in the larval gut (Fig. 1b; Supplementary Fig. 1c–d and Supplementary Video 1).

To exclude the possibility that the food dye employed may have served as an attractant for larvae, 25 starved larvae were presented with stained eggs and an identically coloured piece of agar on the same petri plate (Supplementary Fig. 1e). The larvae selectively aggregated around conspecific eggs and consumed them. We quantified the number of dyed-gut larvae that aggregated around stained eggs and compared this number to the dyed-guy larvae that that aggregated around the agar piece. A significant number of dyed-gut larvae were observed to aggregate around the stained eggs within 30 minutes which further increased significantly after 180 minutes (Supplementary Fig. 1f). These experiments conclude that consumption of conspecific eggs by starved larvae unequivocally involves sensing the nutritional value of eggs.

Next, we evaluated the nutritional benefits of unstained eggs consumed by starved larvae. Towards this end, starved Canton-S larvae reared on nutrition deficient medium were fed synchronized unstained eggs of *w*^1118^ strain. Since *w*^1118^ mutants have white eyes, the Canton-S adults that developed from the starved larvae could readily be distinguished from those that developed from *w*^1118^ eggs. Cannibal larvae showed similar degree of aggregation around the unstained *w*^*1118*^ viable eggs and effectively consumed them (Fig. 1c,d). Once these larvae developed, presence of only red-eyed adults indicated that they consumed all the *w*^*1118*^ eggs. Egg cannibalism was observed regardless of whether stained or unstained eggs were used as source of nutrition (Fig. 1a–d). These results indicate that larvae feeding on eggs successfully pupated and hatched into healthy adults. To determine if these larvae had developed into normal adults, we compared morphological features of both male and female adult flies hatched from both larval groups (i.e., egg fed and standard cornmeal media fed). Adult males and females developed from larvae solely fed on eggs showed no morphological difference when compared to their cornmeal fed counterparts (Fig. 1e–h). We further quantified body weight, wing and body size of adults from each group and found no significant difference (Supplementary Fig. 2). In addition, no significant difference was observed in the number of pupae and adults that developed from larvae consuming conspecific eggs as compared to those that were reared on standard lab media (Fig. 1i–l). Larva-to-pupa and pupa-to-adult transition times were similar in the two groups (Fig. 1j,l). None of the starved larvae survived beyond 24 hours in the negative control which were provided no nutrition at all indicating that the cellulose in Kimwipe® failed to serve as a nutrient. Taken together, these results suggest that *Drosophila* eggs contain essential nutrients required for normal development.

**Figure 2:**
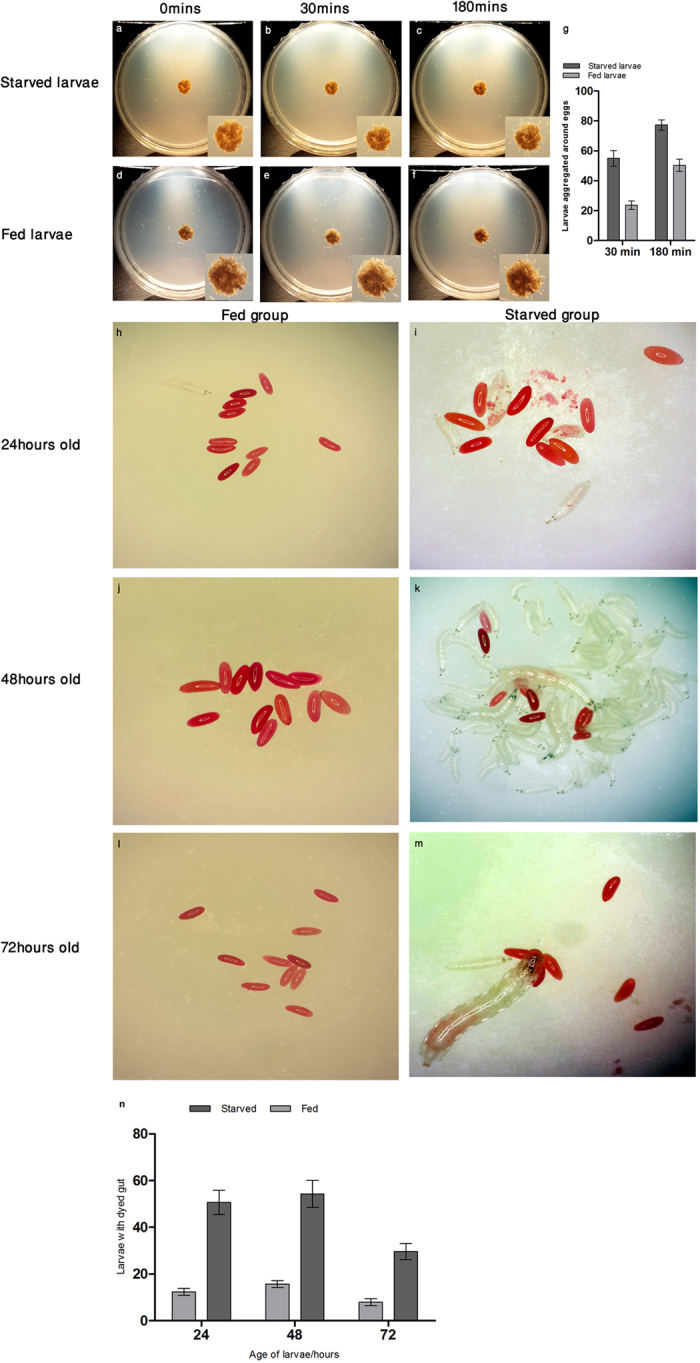
Egg cannibalism is induced by nutritional stress. Comparison of dietary preference for eggs between (**a–c**) starved and (**d-f**) well-fed larvae. Plates were photographed at 0 minutes (**a**,**d**), 30 minutes (**b**,**e**) and 180 minutes (**c**,**f**) after the introduction of larvae. (**g**) Significant difference between fed and starved larval aggregation (mean ± s.e.m.) around eggs was observed at 30 minutes (P = 0.0412) as well as at 180 minutes (P = 0.0155), using paired *t*-test. (**h–m**) Three larval populations (24, 48, 72 hours old) each comprising of two groups (fed and starved) were presented with stained eggs. (**n**) Stained egg consumption, as evidenced by dye accumulation in larval gut, was quantified for all populations. Significant difference was observed between the three populations (P = 0.0105), using one-way ANOVA. Tukey’s post-test analysis showed non-significant difference in larval aggregation between 24 and 48 hours, however, this difference was significant between 24 and 72 hours as well as for 48 and 72 hours.

Next, we investigated whether such cannibalistic feeding behaviour is starvation induced, or are larvae fed on a standard cornmeal medium also capable of cannibalizing their eggs. To address this question, we developed a quantitative assay where a cohort of 100 starved or well-fed larvae were placed in nutrition-deficient media in separate petri dishes. Each petri plate was subsequently supplemented with an equal mass of conspecifics eggs. The degree of aggregation for well-fed (cornmeal fed larvae) and starved larvae was quantified at 30 and 180 minutes after addition of conspecific eggs to each plate (Fig. 2a–g). Starved larvae aggregated around eggs at both time points (Fig. 2b,c). Some aggregation was also observed for well-fed larvae at 30 minutes (Fig. 2e); such larvae however showed increased aggregation around conspecific eggs at 180 minutes (Fig. 2f). Significant differences were observed between well-fed and starved larval aggregation around eggs at 30 and 180 minutes (Fig. 2g). Earlier reports have shown that starvation time for larvae under nutritional stress is 3–4 hours^11^,^13^ which corroborates well with our observation that well-fed larvae aggregate around eggs after 3 hours under nutritional stress thereby suggesting that egg cannibalism is a consequence of starvation.

We further investigated whether larval developmental stage and/or size impact cannibalistic behaviour? The cannibalistic response of starved and well-fed larvae having different age and size was separately assayed and quantified. Larval populations that were 24 hours (1^st^ instar), 48 hours (2^nd^ instar) and 72 hours (3^rd^ instar) of age were obtained from standard laboratory culture of Canton-S. A total of two hundred larvae were derived from each population and further divided into two groups of 100 larvae each. While one group was kept on standard cornmeal media for an additional 4 hours, the other group was starved for the same duration. Subsequently, each group was presented with stained conspecific eggs, and larvae with dyed guts were counted after 30 minutes (Fig. 2h–m). The 1^st^ and 2^nd^ instar well-fed larvae displayed no significant cannibalistic behaviour. Moreover, 1^st^ and 2^nd^ instar starved larvae display similar cannibalistic responses with no significant difference (Fig. 2n). However, the number of dyed-gut larvae dropped significantly in 3^rd^ instar starved population as compared to 1^st^ and 2^nd^ instars. Taken together, no variation in cannibalistic tendency was observed between 1^st^ and 2^nd^ instar starved larval populations suggesting that cannibalistic behaviour does not depend on developmental stage or size of feeding larvae.

Next, we determined if larval preference for food changes after feeding strictly on conspecifics eggs. For that, 1^st^ instar larvae were reared on conspecific eggs for 2 days followed by transfer to a petri plate containing an alternating assortment of cornmeal medium and eggs (Supplementary Fig. 3a-b). Significant larval aggregation was observed around cornmeal medium suggesting that within the same generation larval preference for cornmeal medium does not change after consuming conspecific eggs (Supplementary Fig. 3c). Tendency of starved larvae to feed on conspecific pupae was also investigated. Although, such larvae consumed conspecific eggs (Supplementary Fig. 4a), they failed to consume conspecific *w*^*1118*^ pupae (Supplementary Fig. 4b); larvae also aggregated around pupae but they did not consume them. The subsequent eclosion of only white-eyed adults was attributed to failure of larval consumption of conspecific pupae. After eclosion, larvae continued to aggregate around puparium remains (Supplementary Fig. 4c).

### Starved *Drosophila melanogaster* larvae exhibit carnivorous behaviour

**Figure 3:**
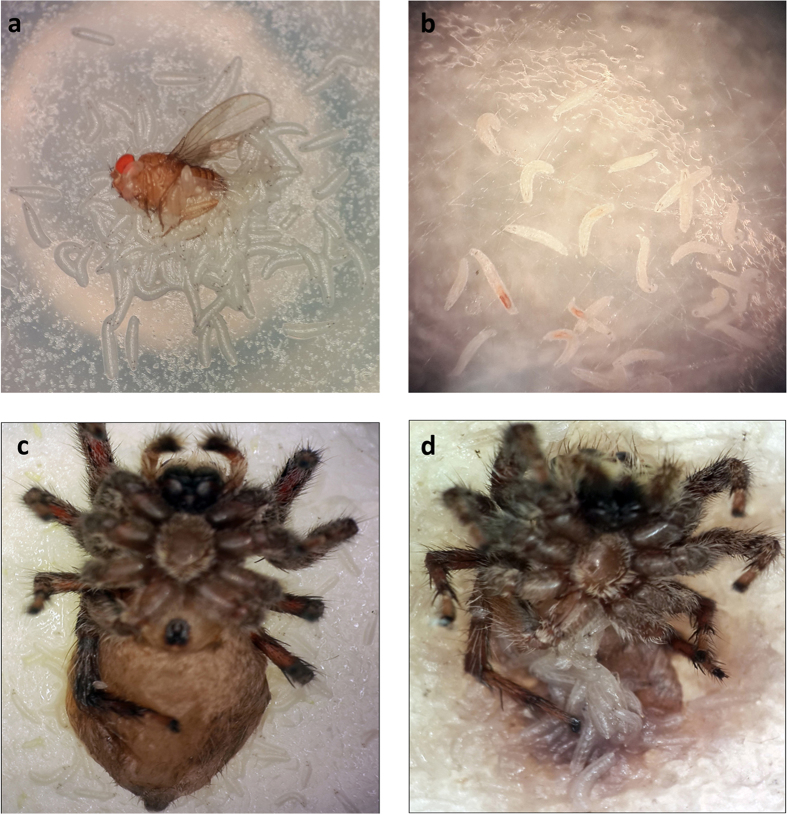
*Drosophila melnogaster* larvae can feed on various carnivorous diets comprising of conspecific and non-conspecific adult carcasses. (**a**) 1^st^ instar larvae scavenging an adult female carcass. (**b**) Adult eye pigment accumulated in larval gut and was observed after larvae were transferred to Phosphate Buffered Saline (PBS) solution. (**c**) 1^st^ instar larvae initiated feeding on a carcass of *Lycosidae*. (**d**) Larval consuption of *Lycosidae* carcass after two days.

Recently it was shown that under nutritional stress *Drosophila melanogaster* larvae can turn into predators and effectively feed on a cannibalistic diet comprising of larger conspecifics^11^. Since *Drosophila melanogaster* larvae are normally known to feed on a non-carnivorous diet and hence are classified as herbivores, we aimed to evaluate the extent of starvation-induced carnivorous behaviour. For this, larvae were reared exclusively on intact conspecific adult carcasses (Fig. 3a) to monitor cannibalistic tendency of larvae. Consumption of adult carcasses resulted in accumulation of *Drosophila* red eye pigment in larval gut (Fig. 3b and Supplementary Video 2). This observation corroborates well with earlier findings where larvae from *Drosophila hydei* were reported to cannibalize conspecific adult carcasses^14^. After observing starvation-induced cannibalism in *Drosophila melanogaster* larvae, we sought to determine if malnourished larvae could feed on carcasses from other taxonomic groups. For this purpose, larvae were reared exclusively on sterilized carcasses of *Musca domestica, Apis mellifera* and *Araneae sp*. In each case, larvae aggregated and consumed the adult carcasses (Fig. 3c,d and Supplementary Fig. 5a-b). Well-fed larvae were also evaluated for their dietary preference with a simultaneous presentation of *Musca domestica* carcass and cornmeal medium (Supplementary Fig. 5c). After 30 minutes of presentation with carcass and cornmeal medium, significant larval aggregation was observed around cornmeal medium as compared to the carcass (Supplementary Fig. 5d-f). These results suggest that starvation induces larvae to feed on a carnivorous diet whereas well-fed *Drosophila* larvae prefer a non-carnivorous diet.

### Starved larvae scavenge conspecific larval carcasses

**Figure 4:**
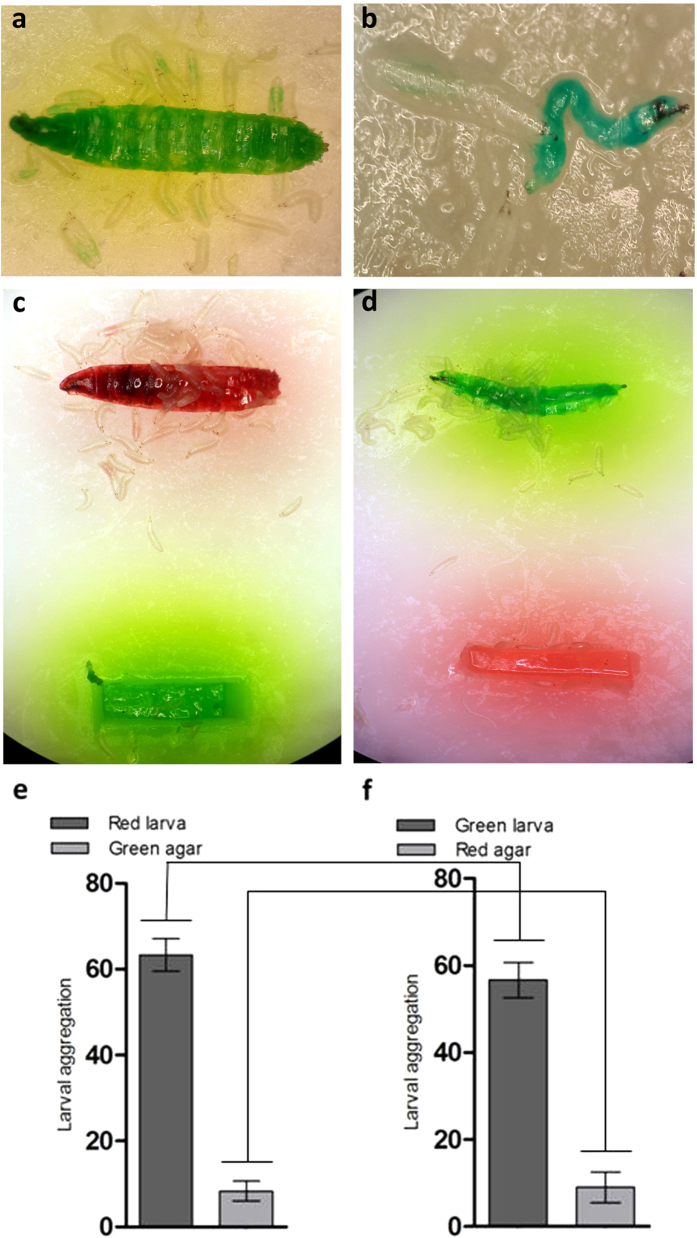
*Drosophila melanogatser* larvae can consume detrivorous conspecific larvae. 1^st^ instar larvae feeding on carcasses of stained (**a**) 3^rd^ instar and (**b**) 1^st^ instar larvae. Dye accumulation can be observed in the gut of feeding larvae resulting from intake of the stained carcass. (**c**) Significant larval aggregation is observed around the red-stained conspecific carcass as compared to the green-agar. (**d**) Larval aggregation persisted around conspecific carcass after swapping dye colors. Quantified data of larval aggregation on (**e**) red carcass and green agar and (**f**) green carcass and red agar. No significant difference was observed in larval aggregation around red or green carcasses (P = 0.1955), using paired *t*-test. Similarly, a non-significant difference was observed in larval aggregation around red and green agar (P = 0.9139), using paired *t*-test.

Younger *Drosophila* larvae have been reported to feed on live wandering-stage conspecific larvae^11^. To evaluate the ability of starved larvae to scavenge conspecific larval carcasses, stained larval carcasses were presented to the starved larvae. As with consumption of stained eggs, starved larvae consumed stained conspecific larval carcasses as evidenced by accumulation of green dye in the larval gut (Fig. 4a,b). We used two different dye colours (red and green) to stain larval carcasses and presented them to starved larvae in the presence of an oppositely coloured piece of agar. One hundred starved larvae were presented with the choice of red coloured larval carcasses and green agar on the same petri plate (Fig. 4c). Another group of 100 starved larvae were presented with the same choice after swapping the colours (i.e. green coloured larval carcass and red agar; Fig. 4d). For each experiment, significant larval aggregation was observed after 30 minutes indicating that starved larvae consumed dead conspecifics due to nutritional content and colour of dye played no role in consumption of larval carcasses (Fig. 4e,f).

### *Drosophila* adults also exhibit cannibalistic behaviour under starvation

**Figure 5:**
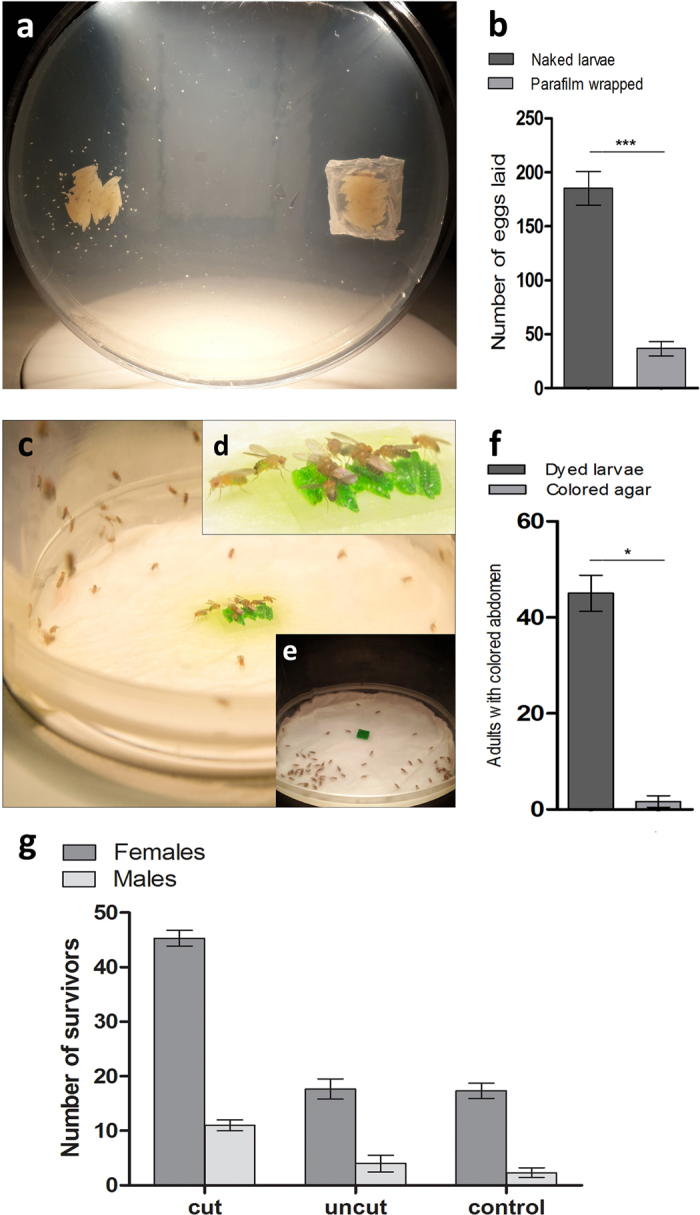
Selective ovipoisitng and cannibalism in *Drosophila melanogaster* adults. (**a**) Flies were assessed for their ovipositing preference between naked and Parafilm covered larval carcasses. Flies preferentially oviposited on and around naked carcasses. (**b**) Eggs were quantified (mean ± s.e.m.) on and around both naked and Parafilm wrapped larvae and a significant preference for naked carcasses was observed (***P = 0.0009), using two tailed student *t*-test. (**c**) Flies exhibit cannibalism when provided with ruptured stained larvae as evidenced by accumulation of green dye in fly abdomens. (**d**) Zoomed-in image of (**c**). (**e**) No fly aggregation was observed on colored agar. (**f**) A significant preference for carcasses (mean ± s.e.m.) was observed (*P = 0.0112) as compared to colored agar, using paired *t*-test. **(g)** Adult flies can only feed on cut/injured carcasses. Quantification of flies starved for 24 hours and exposed to cut or uncut carcasses of conspecific larvae. After continuously feeding for 48 hours, the number of survivors was counted. Equal number of starved flies with no access to any kind of food source served as the negative control. Data represents average of three independent experiments. Degree of variance amongst the three populations (i.e. cut, uncut and control) was evaluated by one-way ANOVA test for males and females separately. The statistics thus obtained were P = 0.0001, P = 0.0043 and F = 101.1, F = 15.43 for females and males, respectively.

The cannibalistic tendency of *Drosophila melanogaster* larvae towards dead larvae led us to question whether adult flies, under starvation, can also feed on carcasses of *Drosophila* larvae. To determine whether such form of cannibalism exists in *Drosophila* adults, starved flies were given intact sterilized carcasses of 3^rd^ instar larvae. Intriguingly, these starving adult flies did not consume cadavers of their conspecific larvae and died within 24 hours. This observation corroborates well with a previous study where starving flies were given access to adult carcasses but no cannibalism was observed^12^. This could be due to the fact that *Drosophila melanogaster* adults lack the essential anatomical structures required to pierce through cuticles of conspecifics^15^. Interestingly, within the same experiment, female adult flies were found to selectively oviposit on and around dead carcasses of conspecific third instar larvae. To further elucidate this behaviour, flies were presented with two groups of larval carcasses: naked or Parafilm® wrapped (Fig. 5a). A significantly greater number of eggs were laid around naked larvae as compared to the Parafilm® wrapped carcasses (Fig. 5b). It is well-known that egg laying in *Drosophila* is directly linked to availability of nutrients^16^ and females prefer laying eggs near nutritious substrates^17^. Our data suggest that although adult flies are unable to consume intact larval carcasses, they preferred to oviposit on and around naked larval carcasses.

To assess if adult flies consider larval carcasses as a potential source of nutrition, starved adults were given access to stained and pierced carcasses of 3^rd^ instar larvae to facilitate feeding. A significant number of adult flies aggregated around and consumed stained carcasses, as compared to coloured agar, as evidenced by accumulation of green dye in fly abdomens (Fig. 5c–f). To measure the viability of starved flies after consumption of carcass contents, we further devised a quantitative assay. Two groups of flies, each having an equal number of males and females (50 males and 50 females), were starved for 24 hours and subsequently presented with 1g of either intact or pierced carcasses. A third group was given no food at all which served as a control. After 48 hours, the number of survivors in each group was counted (Fig. 5g). We observed a significant difference between the numbers of survivors in cages with pierced carcasses as compared to the ones containing intact carcasses. Interestingly, more female survivors as compared to males were observed. Moreover, no significant difference was observed in the number of survivors that were served either intact carcasses or no food (Fig. 5g).

## Discussion

*Drosophila melanogaster* is native to tropical, subtropical and temperate regions of the world^18^, but is also known to inhabit other eco-zones^19^. Considerable variation in fitness-related traits is found across various species of *Drosophila*^20^,^21^,^22^,^23^ suggesting that it might have adapted to a diverse range of environmental conditions such as stress, hypoxia^24^,^25^ and temperature^26^,^27^. In this study, we show that broad and flexible dietary patterns such as egg cannibalism and consumption of carnivorous and detritivorous diets from various sources may also play a vital role in enhancing survival in a wide range of nutrient scarce habitats. According to our findings, the most likely cause of cannibalism in this otherwise non-carnivorous species is to cope with nutritional stress induced by starvation. Under conditions of nutritional stress, larvae that exhibit cannibalistic behaviour have a better chance of surviving as compared to those that are unable to shift to a carnivorous form of feeding. We observed that the development of larvae reared on nutrition scarce media is extremely delayed. Availability of scarce nutrition means that fewer neonates will receive adequate supply of nutrients to develop successfully hence, fewer larvae will survive. Under these conditions, cadavers of dying larvae may serve as an additional food source. One of the most important determinants of cannibalism in *Drosophila melanogaster* is vulnerability of the victim. Immobility and defencelessness of dead conspecifics, eggs and pupae makes them easy targets for cannibalism. Hence, the predator larvae are able to readily feed on untenable victims.

Egg cannibalism is uncommon in *Diphtheria*. So far only one case has been reported in the genus *Tephritidae*^28^ and none in *Drosophilidae*^29^. We show that egg cannibalism exists in *Drosophila melnaogaster* and is induced by nutritional stress. *Drosophila* larvae are sensitive to the types of nutrients present in their diet which primarily include salts, sugars, protein and yeast^30^,^31^. *Drosophila melanogaster* eggs serve as a rich source of such essentials for normal larval development^32^. These include amino acids and sugars^33^, as well as biological macromolecules including proteins, carbohydrates, sugars, lipids and RNA^32^,^34^,^35^. Hence it is not surprising that when starved, larvae can become egg predators and obtain nutritional benefits from them. Not only do *Drosophila* larvae cannibalize conspecifics eggs presented to them in clusters, they are also able to detect and attack single eggs containing minute quantities of nutrition (Supplementary Fig. 4a). Moreover, larvae that feed on conspecifics eggs develop normally. This is supported by the observation that no difference exists in morphological features and development time between larvae that consume conspecifics eggs and those that feed on standard cornmeal lab medium. Based on these observations, we infer that *Drosophila melanogaster* eggs have sufficient nutrition to support normal growth and development. It remains to be determined whether the encounter of potential cannibal larvae with conspecifics eggs is mediated by certain chemical cues similar to those found in certain species that engage in egg cannibalism^36^,^37^,^38^, or is merely an opportunistic feeding behaviour similar to that observed in species like *Hyla pseudopuma* and *Ascia monuste*^39^,^40^.

Asynchronous hatching of eggs can facilitate egg cannibalism. In nature, there exists a non-synchronized population of eggs, as females selectively oviposit^17^,^41^,^42^ repeatedly after assessing environmental factors such as temperature, humidity, light and ethanol^43^,^44^,^45^,^46^. Virgin *Drosophila* females may also lay infertile eggs^47^ which could be consumed by larvae under nutritional stress. Due to asynchronous hatching, larvae that hatch early may encounter non-hatched eggs. If malnourished, these early-hatched larvae may turn cannibalistic towards their non-hatched siblings and have a greater chance of survival. Egg cannibalism may also play a role in regulating population dynamics in nutritionally scarce environments. Larvae feeding on their non-hatched mates may also eliminate upcoming potential competitors for limiting resources. Although *Drosophila* larvae can consume eggs, they fail to feed on conspecifics pupae. While larvae do attack and cluster around conspecifics pupae, they are not able to consume them (Supplementary Fig. 4b).

*Drosophila* females are known to selectively oviposit at suitable egg laying sites only, and may delay egg laying until a suitable site becomes available^44^,^48^,^49^,^50^. Prior to egg laying, females probe potential sites using multiple sensory structures^42^. Our results show that females selectively lay eggs on and around cadavers of conspecific larvae but not around larvae wrapped with parafilm, or elsewhere on soft agar. It therefore seems plausible that the egg laying behaviour is most likely mediated by the nutritional evaluation of deposition sites.

Adults are morphologically ill-equipped to cannibalize by puncturing, as their mouths only have a sucking proboscis, confined to the uptake of food via sopping up liquids^15^. Hence, *Drosophila* adults are considered innocuous consumers of herbivorous food sources and are not known to feed on a cannibalistic diet^12^. However, our findings show cannibalism in *Drosophila melanogaster* is not confined to larvae alone. Adult flies can efficiently feed on carcasses of conspecifics if certain physical barriers are removed. Interestingly, our data show that the number of female survivors, when given access to uncut larvae, was significantly higher than their male counterparts. One plausible explanation for this significant difference between male and female survivors could be presence of higher glycogen reserves in females^51^. Consistent with previous report^51^, we thus conclude that the resistance of females towards starvation was greater than that of males.

Cannibalism in the otherwise non-carnivorous insects is unlikely only to be confined to artificial laboratory conditions and may have broad ecological and evolutionary implication(s)^29^. Our results indicate that *Drosophila melanogaster* can serve as an important behavioural model and help address important and hitherto unanswered questions related to cannibalism, detritivory and carnivory in insects.

## Materials and Methods

### Fly stocks

Wild type Canton-S and *w*^*1118*^ flies were obtained from Bloomington Stock Centre (Bloomington, Indiana University, Indiana, USA). All fly lines were raised on standard cornmeal medium^52^ at 25 °C under a 12 hour (hr) light: 12 hr dark cycle. Behavioural assays employed *w*^*1118*^ eggs and Canton-S larvae.

### Staging of flies for egg collection

In order to collect eggs of relatively similar ages or at same developmental stages (hereafter referred to as synchronized eggs), we followed a previously described staging method^53^. Two days prior to the collection of eggs at relatively similar age, 2–3 days old flies were kept in collection cages containing egg collection containers which were 10–15 cm in diameter. These containers were filled with apple juice/agar-based medium composed of 25% (v/v) apple juice, 1.25% (v/v) glacial acetic acid (Sigma-Aldrich, Cat# 27225) and 2.25% (w/v) bacteriological agar (Sigma-aldrich, Cat# A5306) in 800 ml water. On the day of egg collection, a fresh collection container was added to each fly cage and replaced twice at an interval of 30 minutes to eliminate non-synchronous eggs. Synchronized eggs from the third collection container were dislodged by washing with a smooth paintbrush and transferred to a small egg collection basket.

### Staining of *Drosophila melanogaster* eggs and larvae with food dye

*Drosophila* eggs were stained with the synthetic food dye Tatrazine NaCl. Eggs were dechorionated in a 3% (v/v) sodium hypochlorite solution (Scharlau). Dechrionated eggs were incubated for 2–3 minutes in a silica based desiccation chamber. Subsequently, eggs were transferred to a 5% (w/v) food dye solution and incubated at 4 °C for 16 hours. Eggs were washed with distilled water and placed on 2% (w/v) agar plates (Supplementary Fig. 1a). Stained eggs were collected by visualizing under a stereomicroscope and stored at 4 °C until further use. Similarly, 3^rd^ instar wandering stage larvae of *w*^*1118*^ strain were collected using a wet paintbrush. These larvae were given 3 washings with distilled water and starved for 4–6 hrs in water, to ensure that their gut was cleared of any ingested food. These starving larvae were transferred to eppendorfs containing 5% (w/v) food dye solution and kept at 60 °C for 1 hour. This was followed by incubation at 4 °C for 24 hours and visualized under a stereomicroscope (Supplementary Fig. 1b).

### Assay for egg cannibalism in *Drosophila* larvae

*Drosophila* larvae were exposed to nutritional stress for 3 days prior to presentation of stained *Drosophila* eggs. In order to induce nutritional stress, 30 larvae (hatched from synchronized Canton-S eggs) were reared on nutrient scarce medium composed of 4% (w/v) sucrose (BIO BASIC INC), 1.5% (w/v) bacteriological agar (Sigma-Aldrich), 0.5% (w/v) propionic acid (Apex^TM^) and Nipagin (1 g/lit). A control population of 30 larvae was obtained from the same batch of synchronized eggs and maintained on standard cornmeal medium. Once larvae in both vials started foraging, 10 stained eggs were added to each vial. Larval consumption of eggs was observed under a stereomicroscope at regular intervals for up to 3 hours. Larvae were photographed after they started to aggregate around the eggs.

### Assay for quantifying cannibalism

To quantify the cannibalistic response and rule out role of dye color, 25 starved larvae were presented with a choice between dyed eggs and coloured agar (equal mass) on a petri plate. 4% (w/v) of food dye was used for staining eggs and agar. Larval aggregation on both substrates was observed and photographed at 30 and 180 minutes each. Dyed-gut larvae were quantified on eggs, agar and rest of the plate. The experiment was repeated 3 times and the results averaged. Paired *t*-test was applied to the average number of dyed-gut larvae found on both substrates and elsewhere on the plate at 30 and 180 minutes.

### Assay for development of egg-cannibal larvae

A humid chamber was used to observe the developmental time of larvae feeding solely on conspecific eggs. The chamber was setup by placing light-duty wipes (Kimwipe®) soaked in 3 ml of Nipagin (1 g/L) and 0.5% (v/v) propionic acid. Synchronous eggs were placed at the bottom of a sterilized cylindrical vial (diameter: 5 cm, height: 10 cm) and capped with foam plugs. Synchronous eggs of *w*^*1118*^ flies were collected and placed on a 2% (w/v) agar plate. These plates were stored at −80 °C for 24 hours to ensure that none of the eggs remained viable.

The plates were subsequently transferred for incubation at 25 °C for one hour and 1 g of eggs were transferred to the humid chamber and spread evenly. For the control experiment, 1 g of cornmeal was added to a second humid chamber. A third humid chamber, without any food, served as negative control. 100 first instar Canton-S larvae were transferred into each hydration chamber. These chambers were kept at 25 °C for the larvae to develop. Vials were observed daily and the number of pupae and adults emerging in each vial were counted. This experiment was conducted 3 times and the number of pupae and adults hatched were averaged. Fisher’s exact test was performed on the averaged data from both populations. After 4–6 hours of eclosion, adults were photographed and visually inspected for anatomical differences using a stereo-microscope. An equal number of male and female flies were randomly selected from both populations and their mass, wing and body size determined. To measure wing length, right wings were dissected from the selected flies and mounted on microscope slides secured with glass cover slips. Measurements were taken from the intersection of the anterior cross vein to the third longitudinal vein (L3) on the distal end^54^,^55^. Groups of 25 male and female flies were randomly selected from both populations and weighed on a Mettler Toledo (PB303-s) balance (sensitivity: 0.01 mg). Weight, body and wing length measurements were replicated twice and averaged for each fly. Paired *t*-test was applied to compare the means.

### Assay for starvation as a driving factor of cannibalism in larvae

2 groups of 1^st^ instar larvae, each containing 100 larvae, were used to access starvation driven cannibalism. One group was starved while the other was fed on cornmeal medium for 3 to 4 hours. Each group was then placed on separate petri plates containing nutrition scarce medium. Larvae were allowed to forage for 1 hour at which point both plates were supplemented with 0.5 grams of eggs at the centre of the plate. Larval aggregation around eggs on each plate was photographed and quantified at 30 and 180 minutes. The experiment was repeated 3 times and the results were averaged. Paired *t*-test was applied to the average number of aggregated larvae (fed and starved) at 30 and 180 minutes.

### Quantification of egg cannibalism at different larval stages

3 larval populations were obtained from cornmeal reared Canton-S cultures. Each population consisted of 200 larvae, which were sub-divided into two equal groups. One group was kept on cornmeal medium for 4 hours while the other was starved for the same duration. Subsequently, both groups were presented with dyed eggs on a petri plate with nutrition scarce medium. Dyed-gut larvae were photographed and quantified after 30 minutes. The experiment was repeated 3 times. One-way ANOVA and Tukey’s post-test was performed to determine population variations in the three groups.

### Assay for food preference in egg-fed larvae

25 first instar larvae were reared for two days on eggs. 2% (w/v) agar plate containing alternating patches of cornmeal and eggs was presented to egg-fed larvae (Supplementary Fig. 3). Each patch weighed 0.02 g. Larvae were introduced at centre of the plate and aggregation on each patch was quantified after 30 minutes. The experiment was repeated 3 times and larval aggregation averaged. Two-way *t*-test was applied to compare the means of larvae aggregated on and around both food sources.

### Assay for non-conspecific carnivorous behaviour of larvae

Four groups of 1^st^ instar larvae, each comprising of 30 larvae, were placed in 4 separate vials, which contained 2% (w/v) agar. After 30 minutes of introduction of larvae into the vials, sterilized carcasses of *Drosophila melanogaster, Musca domestica*, *Apis mellifera and Lycosidae spp* were placed in them. Vials were kept at 25 °C and closely observed under a stereomicroscope at regular intervals of 30 minutes. Larvae were photographed once they started to aggregate around the carcasses.

### Assay for larval preference for carnivorous and non-carnivorous diets

A population of 50 cornmeal fed 1^st^ instar larvae was presented with a choice between carnivorous (sterilized *Musca domestica* carcass) and non-carnivorous (cornmeal) diets of equal weight on a 2% (w/v) agar petri plate. The food sources were placed diametrically opposite to each other on the same plate. Larvae were introduced in the centre of the plate (Supplementary Fig. 5c). Larval aggregation was photographed and quantified after 30 minutes. The experiment was repeated 3 times and data averaged. Two-way *t*-test was applied to compare larval aggregation.

### Assay for dye-colour role during conspecific consumption

100 1^st^ instar larvae were presented with a choice of red coloured larval carcass or green coloured agar (of equal weights). Another replicate was run after switching the dye colours (green larvae and red agar). Larval aggregation was photographed and quantified after 2 hours for both replicates. The experiment was repeated 3 times and results averaged. Paired *t*-test was performed to compare larval aggregation on and around red and green carcasses. Another paired *t*-test was performed to compare larval aggregation on red and green agar.

### Assay for ovipositioning preference in flies

A group of 100 adult virgin females and 50 males were raised on cornmeal. An equal number of naked and Parafilm® wrapped larval carcasses were presented in a diametrically opposite sides on a 1.8% (w/v) agar petri plate. The adults were allowed to mate and oviposit for 24 hours at 25 °C. Eggs laid within a 7 mm radius of carcasses were counted. The experiment was repeated 3 times and results averaged. Two tailed *t*-test was applied to compare the number of eggs oviposited on and around naked and wrapped carcasses.

### Assay for cannibalism in adult flies

50 stained 3^rd^ instar carcasses were cut along the dorsal-ventral axis. The cut carcasses were placed in a petri plate lined with moist Kimwipes®. 50 intact carcasses were separately placed in another Kimwipe® lined petri plate. An empty Kimwipe® lined petri plate served as control. The three petri plates were placed in three separate fly cages at 25 °C. 3 days old Canton-S flies were obtained and segregated into males and females. Each population of males and females was starved for 24 hours in vials containing Kimwipes® at 25 °C. Three sets of starved flies, each comprising of 50 males and 50 females, were anaesthetized using CO_2_ and transferred to each fly cage mentioned above. After 48 hours, viable flies in each fly cage were counted. The experiment was repeated 3 times and results averaged. One-way ANOVA and Tukey’s post-test analysis was performed to compare number of flies alive in each fly cage.

To test for a bias towards dye-colour, two groups of 100 adults each (50 males and 50 females), were starved for 24 hours. Subsequently, one group was presented with 50 dyed and cut carcasses while the other was presented with coloured agar (of equal mass). Flies were left for 12 hours at 25 °C. Flies with dyed abdomen were counted within each group. The experiment was repeated 3 times and results averaged. Paired *t*-test was applied to compare the number of flies between groups.

## Additional Information

**How to cite this article**: Ahmad, M. *et al*. Starvation-Induced Dietary Behaviour in *Drosophila melanogaster* Larvae and Adults. *Sci. Rep*. **5**, 14285; doi: 10.1038/srep14285 (2015).

## Supplementary Material

Supplementary Information

Supplementary Video S1

Supplementary Video S2
