# Supplementary material for: Starvation-Induced Dietary Behaviour in Drosophila melanogaster Larvae and Adults
Source: Sci Rep. 2015 Sep 24;5:14285. doi: 10.1038/srep14285 (PMC4585850; doi:10.1038/srep14285)
Supplement: Supplementary Information [file srep14285-s1.doc]

**Supplementary Data**

**Starvation-Induced Dietary Behaviour in *Drosophila melanogaster* Larvae and Adults**

Muhammad Ahmad, Safee Ullah Chaudhary, *Ahmed Jawaad Afzal and *Muhammad Tariq

**AFFILIATIONS**

Department of Biology,

SBA School of Science and Engineering,

Lahore University of Management Sciences,

Sector-U, DHA,

Lahore, 54792,

Pakistan

***CORRESPONDING AUTHORS**

**Dr. Muhammad Tariq**

Email: [m.tariq@lums.edu.pk](mailto:m.tariq@lums.edu.pk)

Tel: +92-42-3560-8218

**Dr. Ahmed Jawaad Afzal**

Email: [ahmed.afzal@lums.edu.pk](mailto:ahmed.afzal@lums.edu.pk)

Tel: +92-42-3560-8354

**
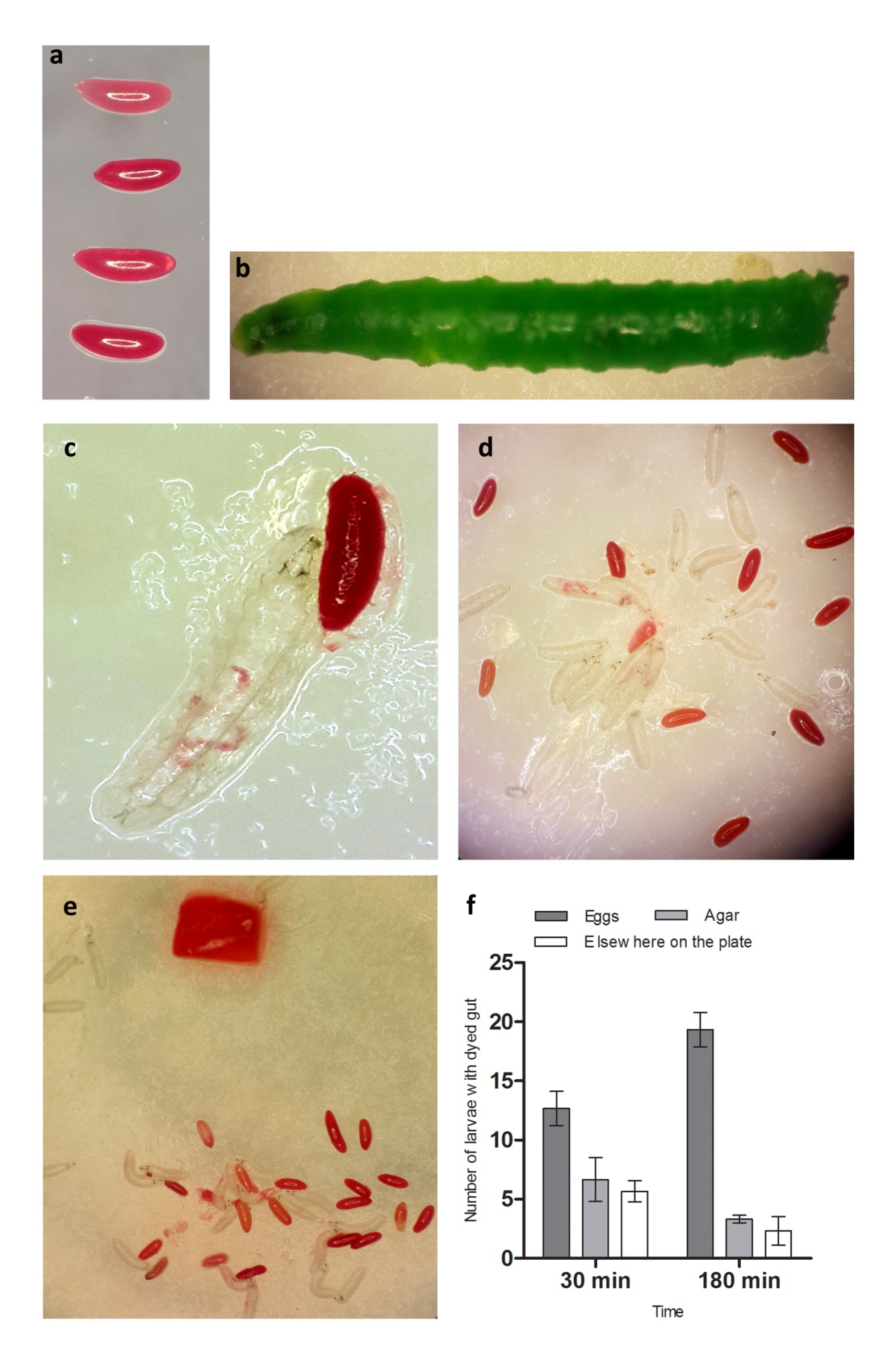
**

**Supplementary Figure 1. (a)** *Drosophila melanogaster* embryos stained with 4% (w/v) solution of food dye. **(b)** Third instar larval carcass stained with a 4% (w/v) solution of food dye. **(c)** and **(d)** Larval consumption of dyed eggs evidenced by dye accumulation in their gut. **(e)** Starved larvae preferentially aggregate around and consume stained eggs when compared to colored agar. **(f)** Significant increase in larval preference (mean ± s.e.m.) was observed for eggs as compared to agar between 30 and 180 minutes (***P=0.0001), using paired *t*-test.

**
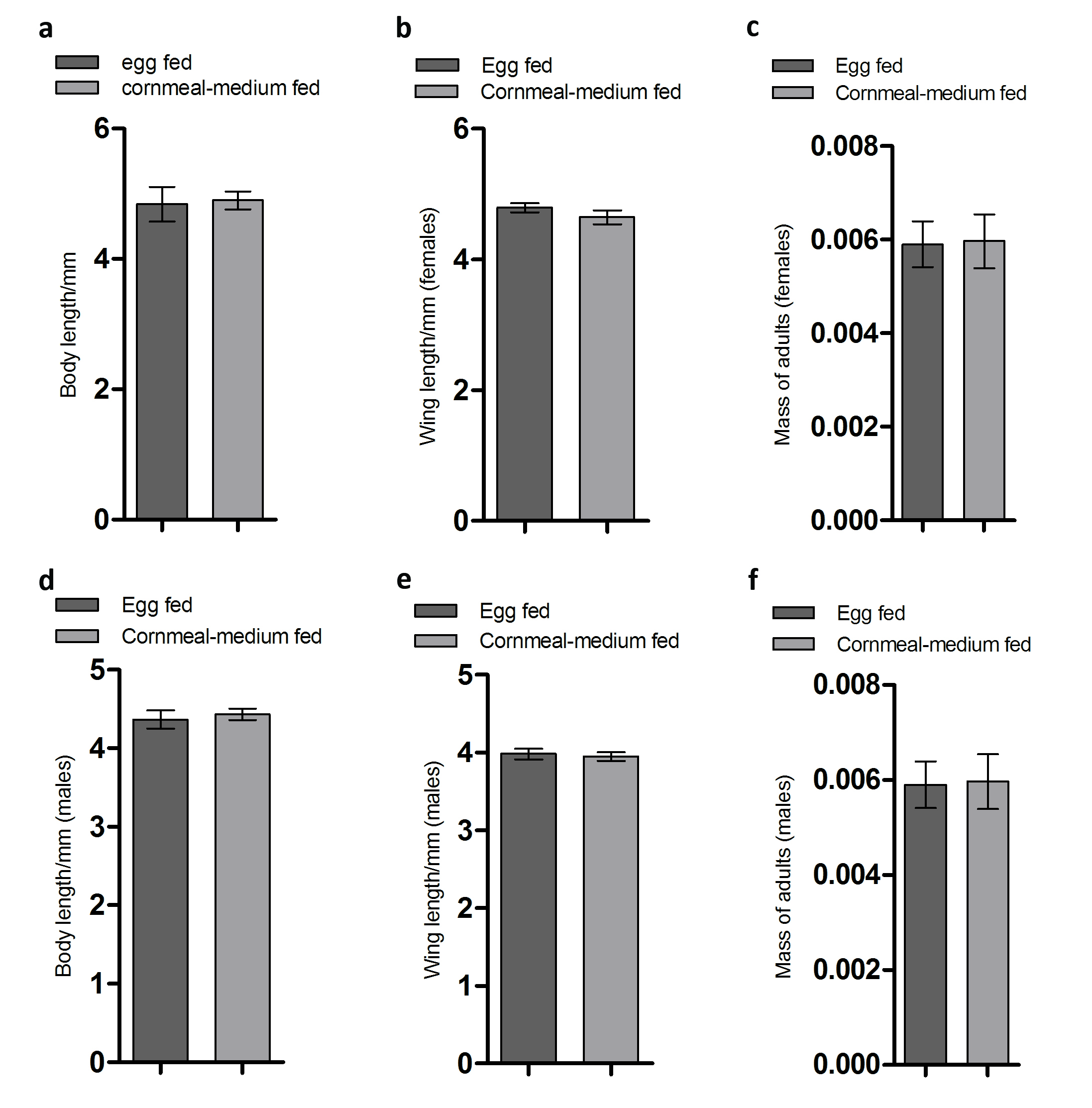
**

**Supplementary Figure 2. *Drosophila* adults which developed from larvae reared on eggs or cornmeal, showed no anatomical differences. (a-c)** Non-significant difference was observed in female (a) body size (P=0.3698), (b) wing length (P=0.1253) and (c) body mass (P=0.5172), using paired *t*-test. **(d-f)** Conformity was also observed in male (d) body size (P=0.6606), (e) wing length (P=0.6304) and (f) body mass (P=0.9514), using paired *t*-tests.

**
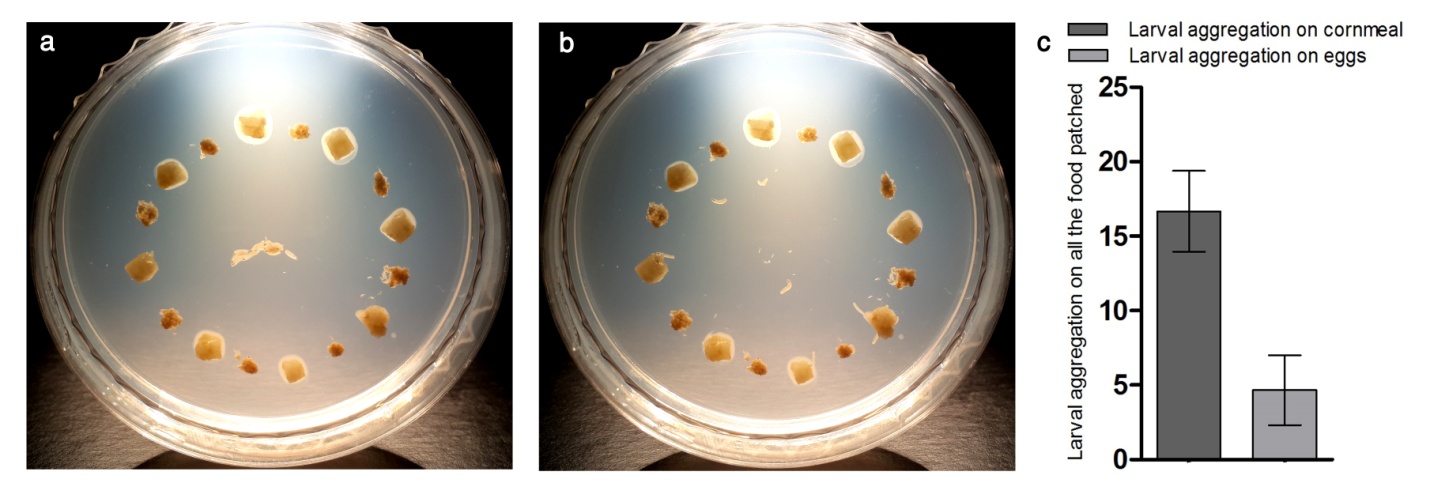
**

**Supplementary Figure 3.** **Larvae prefer cornmeal-medium even after prolonged egg feeding. (a)** 25 Egg-fed larvae visible in the center were presented with a choice between eggs and cornmeal. **(b)** After 30 minutes larvae disappeared within cornmeal food and data was quantified. **(c)** Significant number of larvae aggregated and immersed in cornmeal (*P=0.0288), using two-way *t*-test, as compared to eggs.

**
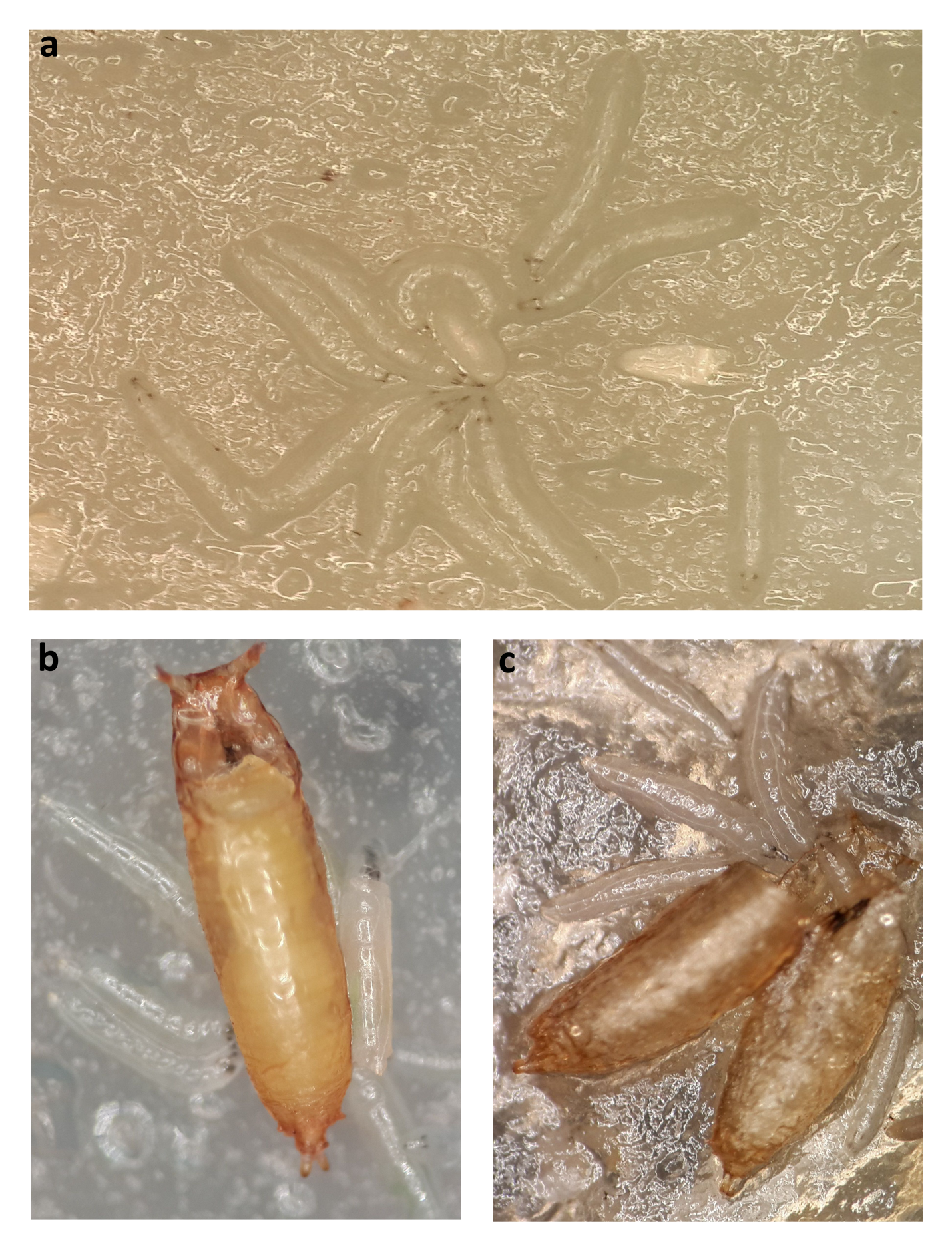
**

**Supplementary Figure 4.** **(a)** Larvae reared on nutrition deficient media, aggregate and attack individual eggs. **(b)** Larvae aggregate around conspecific pupae but fail to consume them. **(c)** Larvae aggregate on puparium after eclosion.

**
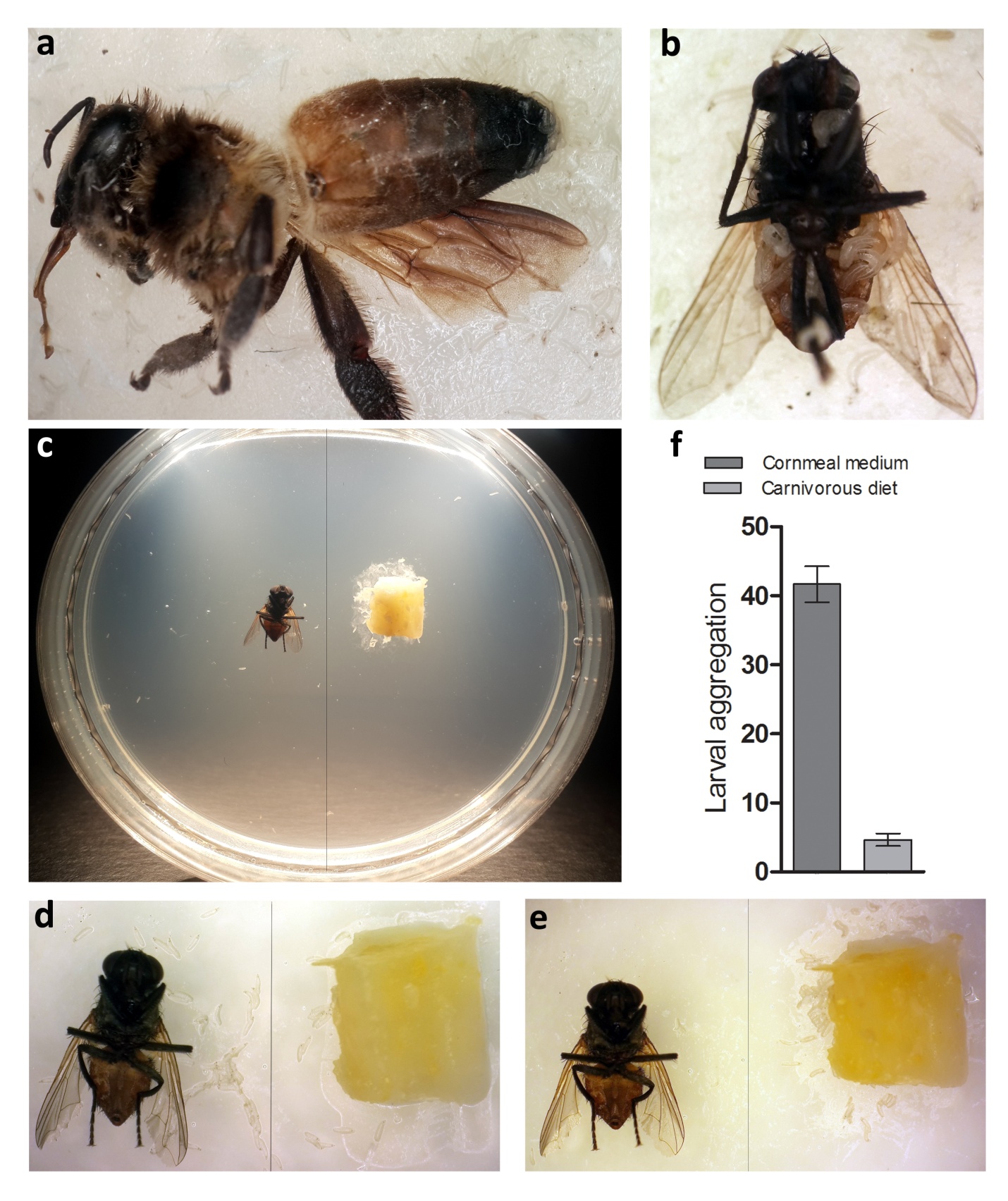
**

**Supplementary Figure 5.** ***Drosophila melanogaster* larvae prefer standard cornmeal medium over a carnivorous diet.** Starved larvae aggregate around **(a)** *Apis mellifera* and **(b)** *Musca domestica* carcases when no alternate choice is available. **(c)** and **(d)** Well-fed larvae placed equidistantly between carnivorous (*Musca domestica*) and non-carnivorous (cornmeal) diets. **(e)** and **(f)** Significant larval aggregation was observed around cornmeal (***P=0.0002), using two-way *t-*test, as compared to the carcass.
